# YEATS2 promotes malignant phenotypes of esophageal squamous cell carcinoma via H3K27ac activated-IL6ST

**DOI:** 10.3389/fcell.2025.1497290

**Published:** 2025-02-18

**Authors:** Yuanfang Zhai, Fanyu Zhang, Xiaoyu Shi, Siwei Zou, Xiaoling Hu, Chengyuan Shan, Ling Zhang, Binbin Zou, Xin Yang, Pengzhou Kong, Xiaolong Cheng

**Affiliations:** ^1^ Key Laboratory of Cellular Physiology of the Ministry of Education (Shanxi Medical University), Translational Medicine Research Center, Department of Pathology, Shanxi Province Cancer Hospital/Shanxi Hospital Affiliated to Cancer Hospital, Chinese Academy of Medical Sciences/Cancer Hospital Affiliated to Shanxi Medical University, Shanxi Medical University, Taiyuan, Shanxi, China; ^2^ Department of Anatomy, Shanxi Medical University, Taiyuan, Shanxi, China; ^3^ Department of Pharmacology, Shanxi Medical University, Taiyuan, Shanxi, China

**Keywords:** ESCC, YEATS2, TAF15, kat5, IL6ST, NF-κB signaling

## Abstract

**Introduction:**

Histone acetylation modifications can regulate gene transcription and play crucial roles in multiple tumorigeneses processes. YEATS domain proteins are one important type of acetylation readers. We have found significant mutations and copy number amplifications of YEATS domain containing 2 (YEATS2) gene in esophageal squamous cell carcinoma (ESCC) through whole genome sequencing (WGS). However, the function and molecular mechanism of YEATS2 in ESCC remain elusive.

**Methods:**

Chi-squared test and Kaplan-Meier methods were used to analyze the clinical significance of YEATS2. MTT, Colony Formation Assay, Transwell, Scratch Wound Healing, subcutaneous tumorigenesis model and lung metastatic tumor model were performed to detect YEATS2 effect on the proliferation and migration ability of ESCC cells *in vivo* and *in vitro* Co-IP-based mass spectrum (MS) assays and Chromatin immunoprecipitation (ChIP) were performed to explore the molecular mechanism of YEATS2 function in ESCC.

**Results:**

ESCC patients with copy number amplification of YEATS2 had shorter postoperative survival. Furthermore, YEATS2 expression was positively correlated with copy number amplification. We have also found that YEATS2 expression was significantly upregulated in ESCC tissues and was correlated closely with the differentiation degree of ESCC cells. The results of in vivo and in vitro experiments revealed that YEATS2 enhanced the abilities of ESCC cells to proliferate and migrate. Mechanistically, YEATS2 activated NF-κB signaling to promote ESCC progression. YEATS2 and H3K27 acetylation (H3K27ac) were both enriched in the promoter region of IL6ST, which is involved in the regulation of YEATS2 on NF-κB signaling. Additionally, YEATS2 could recruit TAF15 and KAT5 to enhance H3K27ac enrichment in the promoter region of IL6ST to regulate its expression.

**Conclusion:**

In conclusion, YEATS2 might function as a potential driver gene and a potential therapeutic target in ESCC.

## 1 Introduction

Esophageal cancer is one of the commonly diagnosed cancers in the digestive system, with an estimated 511,000 new cases and 445,000 deaths worldwide in 2022. The highest rates are seen in Asia, where China has the highest incidence rates, with an estimated 224,000 new cases (Bray et al., 2024). Esophageal cancer consists of esophageal squamous cell carcinoma (ESCC) and esophageal adenocarcinoma (EAC). Squamous cell carcinoma accounts for majority of esophageal cancers in China, and it exhibits significant regional characteristics (Zhu et al., 2023). The molecular heterogeneity and mechanism complexity of ESCC contribute to the lack of effective detection methods and precise targeted therapies (Lin et al., 2018). The 5-year survival rate for ESCC patients is less than 30% (Allemani et al., 2018). To identify new ESCC driver genes, we have collected 508 pairs of ESCC tissues and adjacent esophageal tissues and conducted whole genome sequencing (WGS) in our previous study and found a set of new significant mutation genes related to ESCC, including YEATS domain containing 2 (YEATS2) (Cui et al., 2020).

The YEATS domain was first identified in Yaf9, ENL, AF9, Taf14 and Sas5 (Le Masson et al., 2003; Kabra and Bushweller, 2022) and has been found in more than 100 proteins ranging from yeast to human (Schulze et al., 2009). There are four YEATS domain proteins in human: YEATS1, YEATS2, YEATS3, and YEATS4 (Schulze et al., 2010). In addition to BRD domain proteins and PHD domain proteins, YEATS domain proteins are the newest members of the histone acetylation reader family (Dhalluin et al., 1999; Zeng et al., 2010).

Nucleosome, an octamer complex composed of four histones and DNA, is the basic subunit of chromatin (Li and Reinberg, 2011; Richmond and Davey, 2003). In eukaryotic cells, nucleosomes are folded into dense high-order chromatin structures (van Holde and Zlatanova, 2007). The tight state of chromatin structure could regulate DNA accessibility. The mechanisms causing chromatin structure changes include posttranslational modifications of histones regulated by chromatin modification enzymes and interactions between DNA and histones regulated by chromatin remodeling complexes (Gerhold and Gasser, 2014; Langst and Manelyte, 2015; Jin et al., 2005). Histone modifications participate in most cellular biological processes, including the initiation and progression of cancer, by regulating gene expression (Garraway and Lander, 2013). For histone acetylation modification, histone acetyltransferases transfer the acetyl group to the terminal group of histones, resulting in the elimination of the positive charge on the amino group of histones. The negative charge carried by DNA facilitates the opening of DNA conformation. Once the nucleosome structure is relaxed, transcription factors and co-transcription factors can easily interact with DNA, which in turn promotes transcription of specific genes (Grunstein, 1997; Kuo and Allis, 1998; Forsberg and Bresnick, 2001; Kurdistani et al., 2004). Histone acetylation reader can identify acetylation sites and conduct molecular signals to trigger various downstream biological results. As a novel histone acetylation reader, YEATS2 has been reported to play an important tumor-promoting role in lung cancer (Mi et al., 2017), hepatocellular carcinoma (Wu et al., 2024) and pancreatic ductal adenocarcinoma (Sheng et al., 2023). However, its role and mechanism in ESCC remain unclear.

In this study, we found that YEATS2 was related to the differentiation degree and prognosis of ESCC patients. In addition, *in vivo* and *in vitro* experiments showed that YEATS2 increased the proliferation and migration abilities of ESCC cells. Mechanistically, YEATS2 enhanced the enrichment of H3K27 acetylation (H3K27ac) on the IL6ST promoter region by recruiting TAF15 and KAT5 to promote the IL6ST transcription and further activated NF-κB signaling pathway.

## 2 Materials and methods

### 2.1 Data source and bioinformatics

WGS data from 508 ESCC patients (Cui et al., 2020) and RNA-sequencing (RNA-seq) data from 155 ESCC patients (Liu et al., 2022) have been collected from our previous ESCC cohort studies. Proteomic sequencing data of 124 ESCC patients was collected from Dr. Li, the protein expression values have been normalized and log_2_-transformed (Liu et al., 2021). Somatic mutation profile and copy number variation of YEATS2 gene were collected and analyzed from our previous WGS data of ESCC.

We have performed paired *t*-test to test the expression of YEATS2 in ESCC tissues and adjacent esophageal tissues. We have performed chi-square test to analyze YEATS2 correlation with patient clinical characteristics. Kaplan-Meier survival was used to analyze YEATS2 correlation with patient prognoses. These analyses have been achieved by SPSS software (26.0).

### 2.2 Cell culture

We have collected ESCC cell lines including KYSE150, KYSE180, KYSE450, TE5 and TE9 in our lab. And these cell lines have been authenticated by using Short Tandem Repeat (STR) analysis. We cultured these cells with RPMI 1640 supplemented with 10% FBS at 37°C, 5% CO2. All experiments were performed with mycoplasma-free cells.

### 2.3 siRNA transfection

siRNAs of YEATS2 and IL6ST were purchased from RiboBio Co. The sequences of siRNAs were as follows: CCT​TCA​TCC​TAG​CTA​TAA​A (YEATS2) and GGA​GCA​ATA​TAC​TAT​CAT​A (IL6ST). We transfected ESCC cells with 50 nM siRNA by using riboFECT CP Transfection Kit (RiboBio Co.). And then we performed real-time quantitative PCR (RT-qPCR) and Western blot to detect gene knockdown efficiency at 72 h after transfection.

### 2.4 Vector construction and transfection

pEZ-Lv105-YEATS2 plasmid was purchased from GeneCopoeia Co. And pCMV3-KAT5-Flag plasmid was purchased from Sino Biological. The coding sequences (CDS) of YEATS2 and TAF15 gene were subcloned respectively into the pcDNA3.1 vector with c-terminal 3 × Flag tag (YEATS2-Flag), which was validated by sequencing. 2 μg YEATS2-Flag and blank vector were transfected into ESCC cells in 6 well-plates using EZ Trans Plus (Life-iLab Co.). RT-qPCR and Western blot were performed to detect overexpression efficiency at 72 h after transfection.

### 2.5 Lentiviral transduction

We constructed stable cell l*i*nes of YEATS2 knockdown by using CRISPR/Cas9 system in KYSE150 and KYSE180 (named as Cas9 sg-YEATS2), and stable cell lines of YEATS2 overexpression by using CRISPR/dCas9-SAM system in TE5 and TE9 (named as dCas9 sg-YEATS2). CRISPR/Cas9 system and CRISPR/dCas9-SAM system were purchased from Shanghai Genechem Co. For positive clone screening, we treated stable cell line strains with neomycin and puromycin (Invitrogen Co.) respectively. And then we performed Western blot to detect the efficiency of knockdown and overexpression respectively.

### 2.6 RT-qPCR

We performed quantitative Real-time PCR (qPCR) to detect mRNA expression of target genes according the manual instructions. First of all, we extracted total RNA by using RNAiso Plus (Takara Co.). Then RNA was reversed into complementary DNA by using PrimeScript™ RT Master Mix (Takara Co.). qPCR was performed using TB Green^®^ Premix Ex Taq™ II kit (Takara Co.). With GAPDH as a housekeeping gene, we normalized the relative expression of genes. We calculated the expression of genes by 2^−ΔΔCT^ formula. The experiment was repeated at least three times with three replications in each group.

### 2.7 Western blot

Firstly, we used RIPA lysis buffer to lyse the cells with ultrasonication on ice. After the concentration was determined by using BCA kit (Boster Co.), 50 μg of protein was uploaded to separate by SDS-PAGE electrophoresis. Then proteins were transferred from SDS-PAGE to PVDF membrane (Millipore Co.). After being blocked, the PVDF membrane was incubated with primary antibodies overnight at 4°C, including YEATS2 (Proteintech Co., 1:1,000), histone 3 (H3) (CST Co., 1:1,000), H3K9 acetylation (H3K9ac) (CST Co., 1:1,000), H3K14 acetylation (H3K14ac) (PTM Co., 1:1,000), H3K18 acetylation (H3K18ac) (PTM Co., 1:1,000), H3K27ac (CST Co., 1:1,000), NF-κB (PTM Co., 1:1,000), TAF15 (Abcam Co., 1:10,000), IL6ST (Santa Cruz Co., 1:1,000), KAT5 (Proteintech Co., 1:1,000), GAPDH (Proteintech Co., 1:5,000). Finally, we incubated the PVDF membrane with secondary antibodies for 2 h at room temperature, and visualized them by using ECL kit (Beyotime Co.). The experiment was repeated at least three times.

### 2.8 Co-immunoprecipitation (Co-IP) and mass spectrum (MS)

Co-IP was used to identify the interacting proteins and performed as previously described (Zhai et al., 2022). Co-IP-based MS assays have been performed in TE5 cells with YEATS2 overexpression to explore the interacting protein participating in the regulation of YEATS2 and H3K27ac on IL6ST. Acetonitrile, dithiothreitol, iodoacetamide, NH_4_HCO_3_ and trypsin were first used to digest gel pieces. The tryptic peptides were dissolved in 0.1% formic acid and separated on an EASY-nLC 1000 UPLC system. Peptides were then subjected to NSI source followed by tandem MS/MS in Q Exactive™ Plus (Thermo Co.) coupled online to the UPLC. The resulting MS/MS data were processed using Proteome Discoverer 2.1. The LC-MS/MS analysis was performed at the PTM Bio (China).

### 2.9 Immunofluorescence (IF)

Immunofluorescence (IF) was used to detect the subcellular colocalization among YEATS2, TAF15 and KAT5. Briefly, 3 × 10^5^ cells were cultured on coverslip in 6-well plate. 4% paraformaldehyde was used to fix the cells at room temperature for 0.5 h. 0.25% Triton100/PBS was used to permeabilize the cells at room temperature for 0.5 h. After being blocked with 5% BSA overnight at 4°C, cells were incubated with primary antibodies cells for 2 h at room temperature and secondary antibodies for 2 h at room temperature away from light. We then counterstained cells with DAPI to label nucleus with blue fluorescence. In the end, we captured ESCC cell pictures by using the laser scanning confocal microscopy.

### 2.10 Animal studies

We have constructed a subcutaneous tumorigenesis model and lung metastatic tumor model in 6-week-old BALB/c nude female mice (Gempharmatech Co.). For subcutaneous tumorigenesis model, we injected subcutaneously 1.5 × 10^6^ cells into nude mice groin and measured the length and width of xenograft tumors every 3 days until 35 days. Finally, xenograft tumors were dissected, photographed, fixed in 10% formalin, embedded in paraffin, and confirmed by HE staining. We used the formula “volume = (length × width^2^)/2” to calculate the size of xenograft tumors.

For lung metastatic tumor model, we injected 8 × 10^5^ cells into tail vein of nude mice. The nude mice were fed continuously for 60 days. The lung tissues were dissected and photographed. And then we used 10% formalin to fix them and paraffin to embed them respectively. In the end, metastatic lesions in the lung were confirmed by HE staining.

### 2.11 Dual-luciferase reporter assay

We used NF-κB-Luc luciferase reporter plasmids to determine YEATS2 effect on the transcriptional activity of NF-κB signaling pathway. 800 ng of NF-κB-Luc plasmid and 32 ng of PRL-Tk plasmid were transfected simultaneously into cells in 24-well-plate. 48 h after transfection, we used luciferase reaction reagent (TRANS Co.) to measure firefly luciferase activity and renilla luciferase activity using luminometer respectively. Finally, we calculate NF-κB reporter activity using firefly luciferase activity/renilla luciferase activity. The experiment was repeated at least three times with three replications in each group.

### 2.12 Chromatin immunoprecipitation (ChIP)

According to User Guide, ChIP assay kit (CST Co.) was used to perform ChIP assay. In order to screen out the genes, on which YEATS2 and H3K27ac were both enriched, antibodies of YEATS2 (Proteintech Co.) and H3K27ac (CST Co.) were used to precipitate the DNA fragments. After being found that YEATS2 and H3K27ac were enriched in the promoter of IL6ST, TAF15-pcDNA3.1-Flag and KAT5-pCMV-Flag plasmids were transfected into cells. Anti-Flag antibody (CST Co.) was used to determine the enrichment of TAF15 and KAT5 on the promoter of IL6ST, and the isotype IgG (Proteintech Co.) was used as a negative control. The experiment was repeated at least three times.

### 2.13 Statistical analysis

All data were presented as mean ± SD. We performed statistical analysis by using SPSS 22.0. And we visualized graphs by using Prism 5 GraphPad. Statistical significance is assessed by *p* value, including *p* < 0.05 (*), *p* < 0.01 (**), *p* < 0.001 (***).

## 3 Results

### 3.1 The clinical significance of YEATS2 in ESCC

**FIGURE 1:**
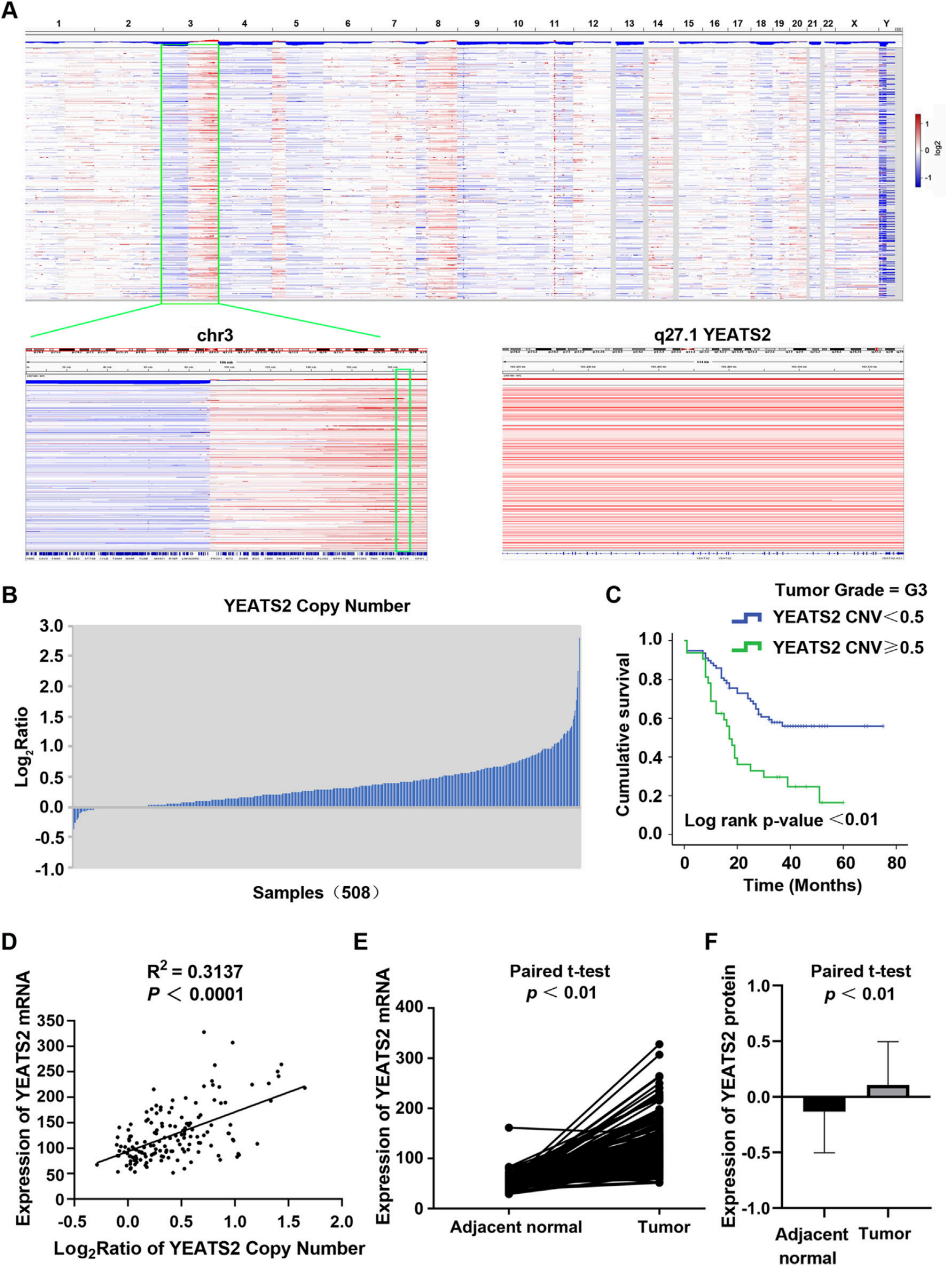
The expression and prognostic value of YEATS2 in ESCC. **(A)** The copy number variations of 508 ESCC tissues were analysed by using GISTIC based on our previous project. **(B)** The copy number amplifications of YEATS2 in 508 ESCC genomes. **(C)** Kaplan-Meier was performed to analyze the associations of the copy number of YEATS2 with postoperative survival time for G3-stage ESCC patients. **(D)** The correlation between expression of YEATS2 mRNA and YEATS2 copy number in 155 ESCC tissues. Statistical significance was assessed by Pearson’s chi-squared test. **(E)** YEATS2 mRNA expression in ESCC tissues and normal esophageal tissues from RNA-seq data of 155 ESCC patients. **(F)** YEATS2 protein expression in ESCC tissues and normal esophageal tissues from proteomic sequencing data of 124 ESCC patients. The protein expression values have been normalized and log2-transformed.

We previously conducted ESCC cohort studies to explore the molecular mechanism of ESCC, which generated WGS data of 508 ESCC patients (Cui et al., 2020) and RNA-seq data of 155 ESCC patients (Liu et al., 2022). From WGS cohort study of 508 ESCC patients, YEATS2 was identified as one of genes to be significantly mutated, with being mutated in 5.31% (27/508) of ESCC cases. Besides, the results of WGS data analysis also identified the copy number amplification (CNA) of the chromosome 3q27 region containing YEATS2 (Figure 1A). And CNA of YEATS2 were identified in 27.56% (140/508) of ESCC cases (Figure 1B). Chi-squared test and Kaplan-Meier methods were used to analyze the clinical significance of YEATS2. The chi-squared test revealed that copy number variation of YEATS2 was significantly associated with ESCC patient differentiation (Table 1). And we have found that ESCC patients in G3-stage with higher copy number of YEATS2 had a worse prognosis, as shown by Kaplan-Meier analysis (Figure 1C).

**TABLE 1:** Relationship between YEATS2 copy number and clinical characteristics of patients.

Characteristics	Total	YEATS2 CNV		*P* value
	*n = 506*	CNV<0.5	CNV>0.5	
Age		366	140	
< 60	218	154	64	0.483
≥ 60	288	212	76	
Gender				
Male	334	239	95	0.602
Female	172	127	45	
Smoking				
never	252	186	66	0.487
light	21	16	5	
moderate	152	106	46	
heavy	81	58	23	
Drinking				
never	345	255	90	0.286
light	73	46	27	
moderate	67	50	17	
heavy	21	15	6	
Differentiation				
High	20	8	12	High/middle+low, 0.003 High+middle/low, 0.076
Middle	310	222	88	
Low	176	136	40	
T Classification				
T1	42	28	14	T1/(T2+T3), 0.374 (T1+T2)/T3, 0.674
T2	125	95	30	
T3	339	243	96	
T4	0	0	0	
N classification				
N < 1	296	215	81	0.92
N ≥ 1	210	151	59	
TNM stage				
I	45	32	13	(I +II+III)/IV, 0.377 (I+II)/(III+IV), 1.000
II	252	183	69	
III	182	129	53	
IV	27	22	5	

Furthermore, integrated analysis of WGS and RNA-seq data revealed that YEATS2 mRNA expression was positively correlated with CNA (Figure 1D). Analysis of RNA-seq data from 155 ESCC patients revealed a significant upregulation of mRNA expression of YEATS2 in ESCC tissues (Figure 1E). Furthermore, we have re-analyzed proteomic sequencing data of 124 ESCC patients from Dr. Li (Supplementary Table S1). The results of proteomic data analysis also showed a significant upregulation of protein expression of YEATS2 in ESCC tissues (Figure 1F). The chi-squared test also revealed that the protein expression level of YEATS2 was significantly related to ESCC differentiation (Table 2). Consequently, we suspected that YEATS2 might act as an oncogene and be related to the differentiation and prognosis of patients with ESCC.

**TABLE 2:** Relationship between YEATS2 protein expression and clinical characteristics of patients.

Characteristics	Total	YEATS2 expression		*P* *value*
	(n =124)	Low(T<0.095)	High(T>0.095)	
Smoking		62	62	
YES	42	24	18	0.255
NO	82	38	44	
Drinking				
YES	14	6	8	0.57
NO	110	56	54	
Tumor location				
Upper	6	1	5	Upper / Middle + Lower, 0.090 Upper + Middle / Lower, 0.145
Middle	75	36	39	
Lower	42	25	17	
Differentiation				
High	17	13	4	High/middle+low, 0.012 High+middle/low, 0.009
Middle	82	40	42	
Low	19	4	15	
T Classification				
T1	2	0	2	(T1+T2)/T3, 0.143
T2	18	7	11	
T3	104	55	49	
T4	0	0	0	
N classification				
N < 1	61	27	34	0.209
N ≥ 1	63	35	28	
TNM stage				
I	6	3	3	I/(II+III), 1.000 (I+II)/ III, 0.281
II	58	26	32	
III	60	33	27	
IV	0	0	0	

### 3.2 YEATS2 promoted proliferation and migration of ESCC cells

WGS data analysis, RNA-seq data analysis and proteomic data analysis have indicated that YEATS2 might promote the progression of ESCC. To explore YEATS2 function in ESCC, we firstly knocked down YEATS2 expression in ESCC cell lines KYSE450 and KYSE180 by transfecting siRNAs targeting YEATS2, and increased YEATS2 expression in TE5 by transfecting over-expression plasmids of YEATS2 (Supplementary Figures S1A, B). We have found that YEATS2 knockdown inhibited the proliferation and migration of KYSE450 and KYSE180. Conversely, YEATS2 overexpression promoted the migration of TE5, but did not affect its proliferation (Supplementary Figures S1E, H).

**FIGURE 2:**
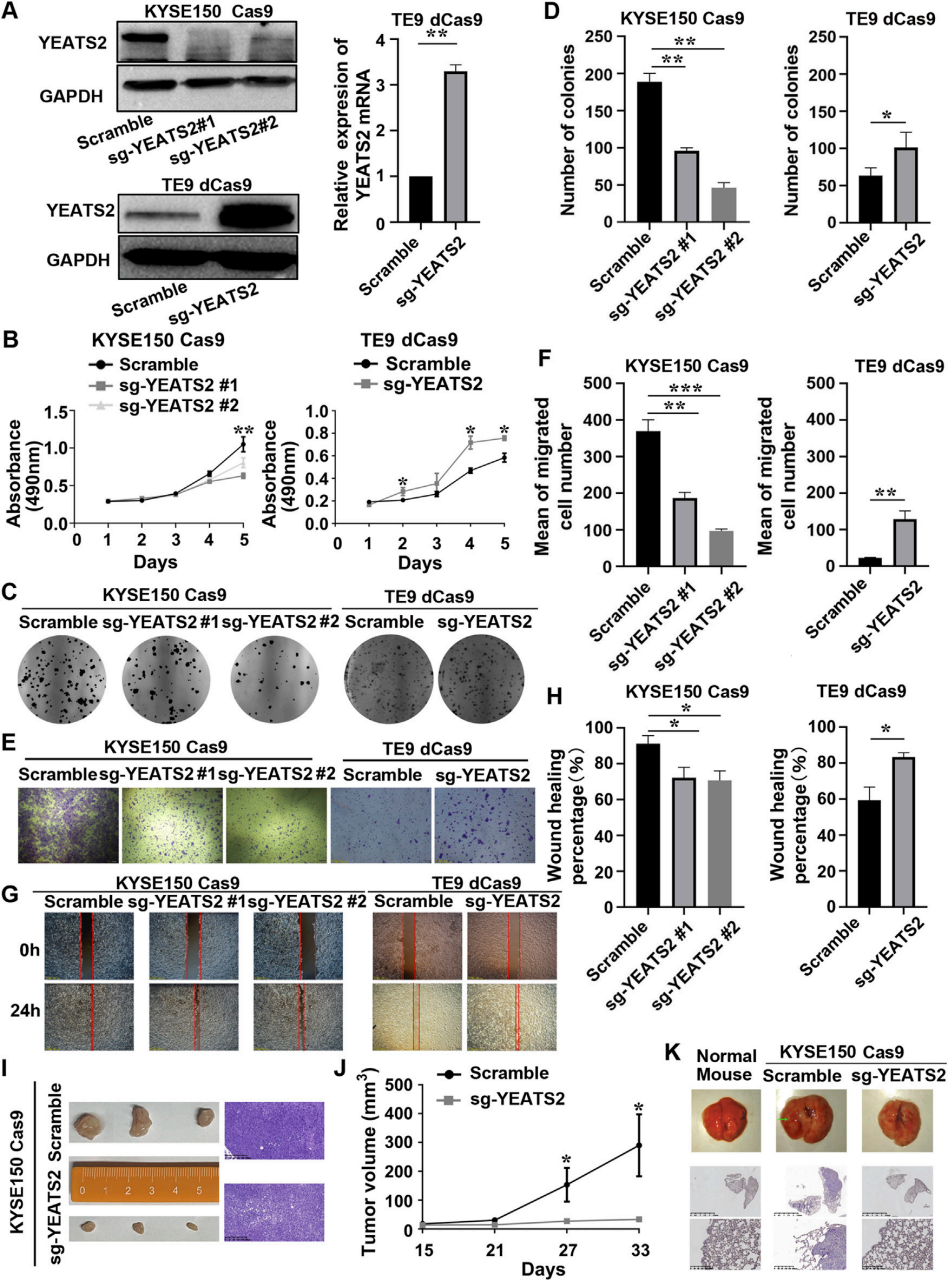
YEATS2 stable knockdown inhibited the abilities of ESCC cells to proliferate and migrate *in vitro and in vivo*. **(A)** Western blot and RT-qPCR were performed to verify the efficiency of YEATS2 knockdown and overexpression acted by CRISPR/Cas9 and CRISPR/dCas9-SAM system. Data shown are the mean ± SD of three biological replicates. *p* value was calculated by unpaired t tests with *p < 0.05, **p < 0.01, and ***p < 0.001. **(B)** MTT was performed to detect YEATS2 effect on the proliferation ability of ESCC cells *in vitro*, including KYSE150 and TE9. Data shown are the mean ± SD of three biological replicates. *p* values were calculated by one-way ANOVA or unpaired t tests with *p < 0.05, **p < 0.01, and ***p < 0.001. **(C–D)** Colony Formation Assay was performed to detect YEATS2 effect on the colony forming ability of ESCC cells *in vitro*, including KYSE150 and TE9. Data shown are the mean ± SD of three biological replicates. *p* values were calculated by unpaired t tests with *p < 0.05, **p < 0.01, and ***p < 0.001. **(E–F)** Transwell was used to detect YEATS2 effect on the migration ability of ESCC cells *in vitro*, including KYSE150 and TE9. Data shown are the mean ± SD of three biological replicates. *p* values were calculated by unpaired t tests with *p < 0.05, **p < 0.01, and ***p < 0.001. **(G–H)** Scratch Wound Healing was used to detect YEATS2 effect on the wound healing ability of ESCC cells *in vitro*, including KYSE150 and TE9. Data shown are the mean ± SD of three biological replicates. *p* values were calculated by unpaired t tests with *p < 0.05, **p < 0.01, and ***p < 0.001. **(I)** Photographic images and hematoxylin–eosin (HE) staining of tumors from nude mice (n = 3 in each group). **(J)** The volume change of tumors from tumor-bearing nude mice. Tumor volume was calculated and diameters were measured at a regular interval of 6 days for up to 33 days. Data shown are the mean ± SD of three biological replicates. *p* values were calculated by unpaired t tests with *p < 0.05, **p < 0.01, and ***p < 0.001. **(K)** Representative images of lungs from tail-vein lung metastatic models (n = 4 in each group) and HE staining for lungs from tail-vein lung metastatic models.

We have found that KYSE150 is more suitable for the establishment of subcutaneous tumorigenesis model and lung metastatic tumor model in nude mice. To further verify YEATS2 function in ESCC cell lines, we established stable cell lines for YEATS2 knockdown by using CRISPR/Cas9 system in KYSE150 and KYSE180, and stable cell lines for YEATS2 overexpression by using CRISPR/dCas9-SAM system in TE5 and TE9 (Figure 2A; Supplementary Figures S1C–D). We have found that stably knocking down of YEATS2 in KYSE150 and KYSE180 inhibited the abilities to proliferate, form colonies, migrate and heal scratch *in vitro*. Conversely, stably over-expression of YEATS2 promoted the abilities to proliferate, form colonies, migrate and heal scratch in TE5 and TE9 (Figures 2B–H, Supplementary Figures S1F–I). And we also reintroduced YEATS2 in KYSE150 Cas9 sg-YEATS2 by transfecting over-expression plasmids of YEATS2 and performed rescue experiment. As expected, reintroduction of YEATS2 rescued the decrease of colony formation and migration abilities inducing by YEATS2 inhibition (Supplementary Figures S2A–C). Besides, by constructing subcutaneous tumorigenesis model and lung metastatic tumor model in nude mice, we have found that stable YEATS2 knockdown significantly inhibited proliferation and migration of KYSE150 *in vivo* (Figures 2I–K; Supplementary Figures S2D–F). Consequently, the results of *in vivo* and *in vitro* experiments showed that YEATS2 enhanced the proliferation and migration abilities of ESCC cells.

### 3.3 YEATS2 activated NF-κB signaling pathway

**FIGURE 3:**
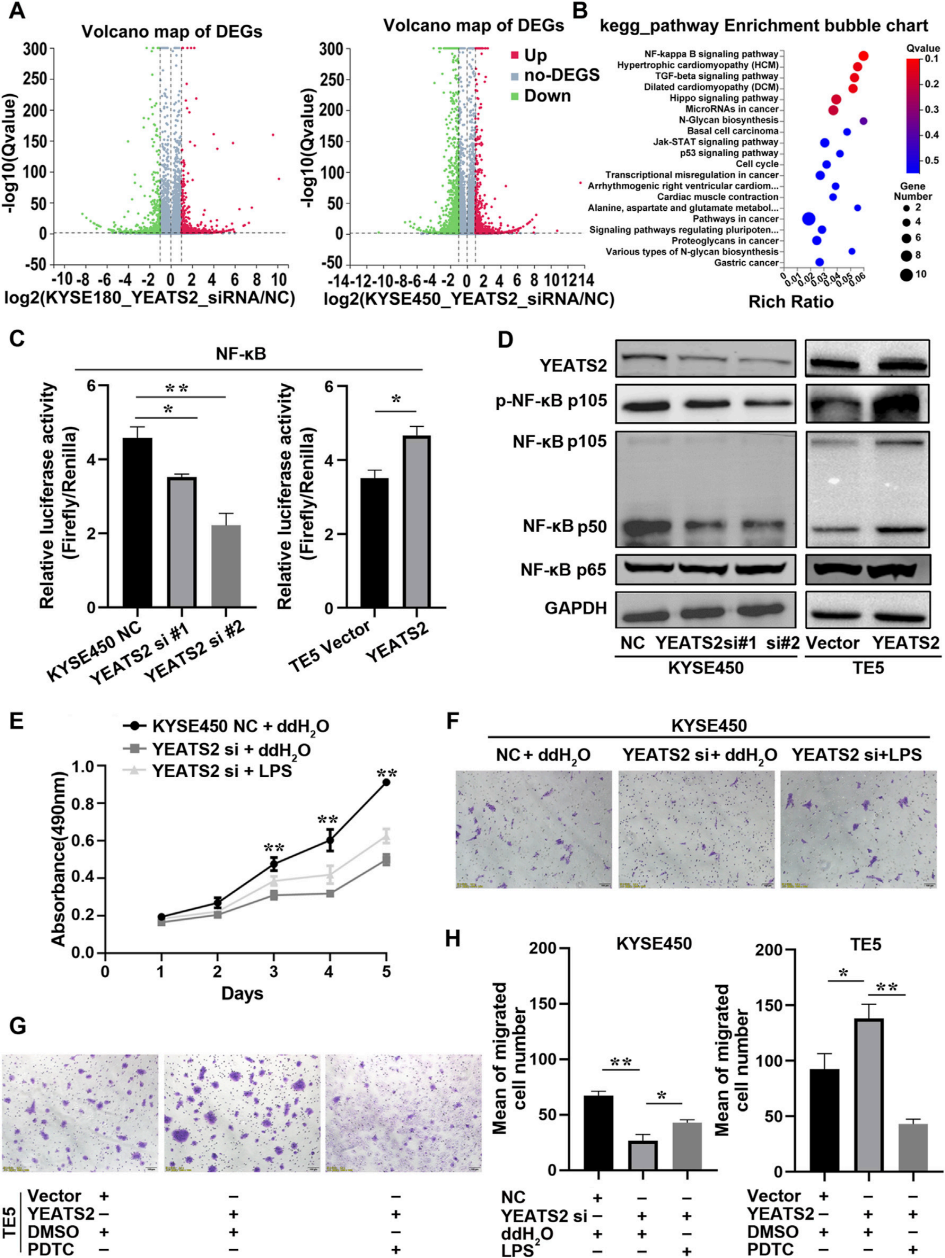
NF-κB signaling pathway mediated YEATS2 function. **(A)** RNA-seq were performed before and after YEATS2 knockdown on KYSE450 and KYSE180 cells. The volcano maps of DEGs in KYSE180 and KYSE450 from RNA-seq. **(B)** The KEGG analyses of 221 DEGs derived from RNA-seq. **(C)** The YEATS2 regulations on the transcriptional activity of NF-κB signaling pathway were verified by Dual-Luciferase Reporter Assay. Data shown are the mean ± SD of three biological replicates. *p* values were calculated by unpaired t tests with *p < 0.05, **p < 0.01, and ***p < 0.001. **(D)** The YEATS2 regulations on NF-κB p105/p50 and p-NF-κB p105 levels were verified by Western blot. **(E, F, H)** LPS weakened the effect of YEATS2 knockdown on the proliferation and migration of KYSE450. Data shown are the mean ± SD of three biological replicates. *p* values were calculated by one-way ANOVA with *p < 0.05, **p < 0.01, and ***p < 0.001. **(G, H)** PDTC weakened the effect of YEATS2 overexpression on the migration of TE5. Data shown are the mean ± SD of three biological replicates. *p* values were calculated by unpaired t tests with *p < 0.05, **p < 0.01, and ***p < 0.001.

To better understand the molecular mechanism of YEATS2 function, we performed RNA-seq on KYSE450 and KYSE180 cells with YEATS2 knockdown (Supplementary Tables S2–S6). After YEATS2 knockdown, there were 1,567 differentially expressed genes (DEGs) (Fold change>2, *p* < 0.001) in KYSE450 and 747 DEGs (Fold change>2, *p* < 0.001) in KYSE180. And 221 genes were differentially expressed in both KYSE450 and KYSE180 (Figure 3A; Supplementary Figure S2G). We also found that the DEGs were obviously enriched in NF-κB signaling pathway through Kyoto Encyclopedia of Genes and Genomes (KEGG) and Gene Ontology (GO) analyses (Figure 3B; Supplementary Figure S2H).

Therefore, we performed dual-luciferase reporter assay and Western blot to detect the correlation between YEATS2 and the NF-κB signaling pathway. We found that YEATS2 promoted the transcriptional activity of NF-κB signaling pathway and the levels of NF-κB p105/p50 and p-NF-κB p105 (Figures 3C, D; Supplementary Figure S3A–B). Besides, LPS, an agonist of NF-κB signaling pathway, partially reversed the inhibitory effect of YEATS2 knockdown on the proliferation and migration of KYSE450 cells (Figures 3E, F, H). Conversely, PDTC, an inhibitor of NF-κB signaling pathway, partially reversed the promotional effect of YEATS2 overexpression on TE5 cells (Figures 3G, H). Consequently, YEATS2 might promote the progression of ESCC through NF-κB signaling pathway.

### 3.4 YEATS2 activated NF-κB signaling pathway by regulating IL6ST

**FIGURE 4:**
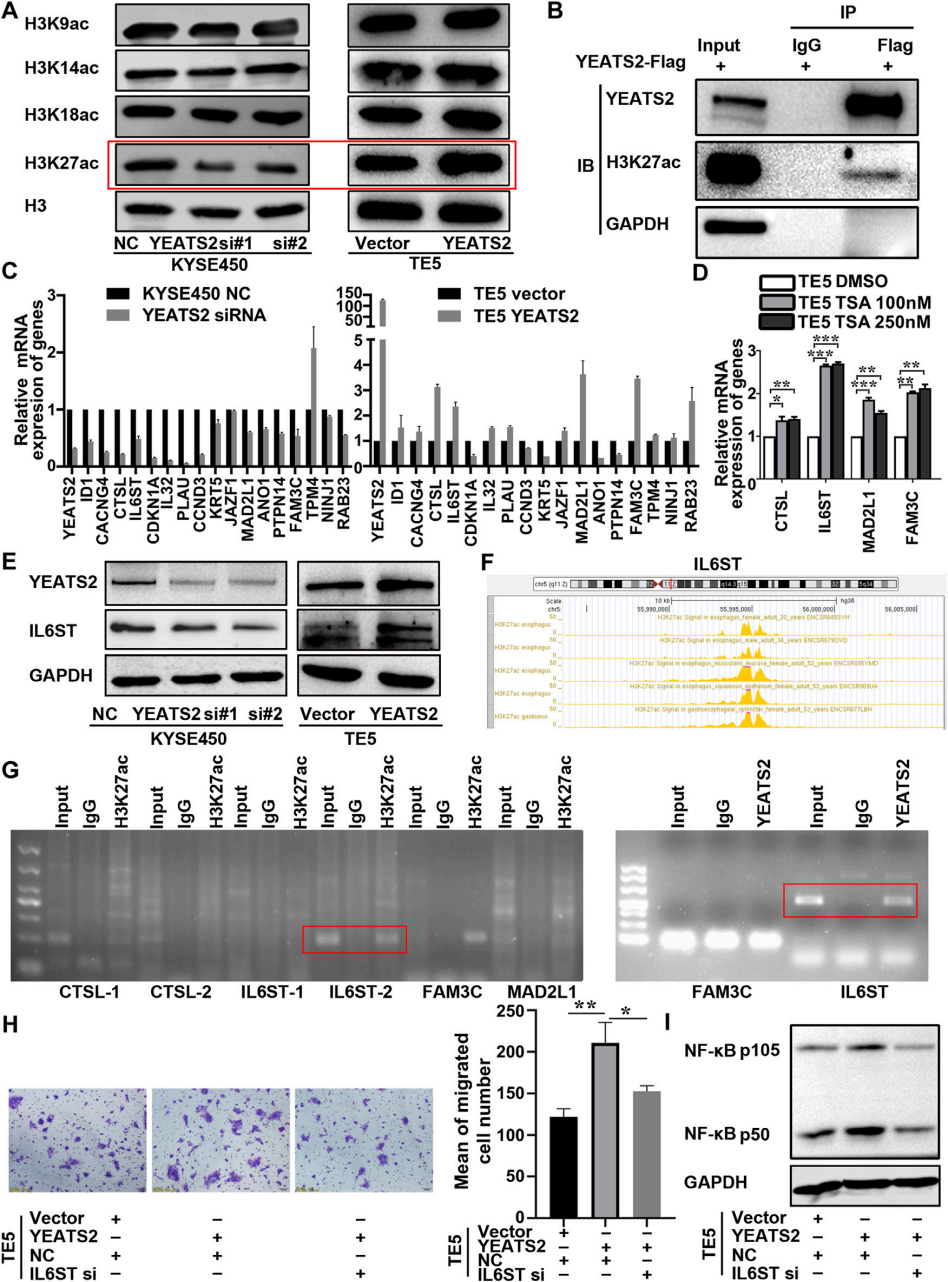
YEATS2 and H3K27ac were enriched in the promoter of IL6ST. **(A)** The YEATS2 relationships with acetylation level of the common lysine sites of H3 histone were detected by Western blot. **(B)** The binding of YEATS2 with H3K27ac was verified by Co-IP. **(C)** YEATS2 and 17 DEGs mRNA expression levels were detected by performing RT-qPCR. **(D)** TSA, a histone deacetylase inhibitor, prominently increased IL6ST expression. **(E)** YEATS2 regulated the protein expression of IL6ST. **(F)** H3K27ac enrichment in the promoter of IL6ST was predicted by the ENCODE database. **(G)** ChIP was used to detect YEATS2 and H3K27ac enrichment in the promoter of CTSL, IL6ST, MAD2L1 and FAM3C. **(H)** IL6ST knockdown partially reversed the effect of YEATS2 on the migration of TE5. Data shown are the mean ± SD of three biological replicates. *p* values were calculated by unpaired t tests with *p < 0.05, **p < 0.01, and ***p < 0.001. **(I)** IL6ST was involved in the regulation of YEATS2 on p105/p50 protein.

In ESCC, the underlying mechanism by which YEATS2 regul*at*es NF-κB signaling pathway remained to be elusive. It has been reported that YEATS domain proteins, as histone acetylation readers, can regulate histone acetylation by recruiting histone acetyltransferases, thereby regulate the transcription of specific genes (Mi et al., 2017). Therefore, we detect YEATS2 correlation with the acetylation of common lysine sites of histone 3. We found that YEATS2 regulated H3K27ac exclusively, but YEATS2 had no effect on the level of H3K9ac (Figure 4A). Besides, the results of Co-IP showed that YEATS2 could interact with H3K27ac in KYSE150 cell (Figure 4B). Consequently, YEATS2 could not only recognize H3K27ac but also regulate it further in ESCC cells.

H3K27ac enrichment in the promoter region of genes is a transcription activation marker (Wang et al., 2024). We selected 17 DEGs related to NF-κB signaling pathway from 221 DEGs to investigate the relationship in mRNA expression level between YEATS2 and these 17 DEGs. And we found that the mRNA expression levels of CTSL, IL6ST, MAD2L1 and FAM3C were positively correlated with YEATS2 (Figure 4C), which was consistent with the RNA-seq data (Supplementary Figure S3C). Further analysis of GSE53625 data revealed that the mRNA expression level of YEATS2 was positively related to IL6ST, MAD2L1, and FAM3C (Supplementary Figure S3D).

Trichostatin A (TSA), a histone deacetylase inhibitor, was firstly applied to test relationship between H3K27ac and 4 DEGs. Result of RT-qPCR showed that TSA significantly increased mRNA expression level of IL6ST (Figure 4D). Consequently, H3K27ac might be involved in the regulation of YEATS2 on IL6ST. And YEATS2 could also regulate the protein expression level of IL6ST (Figure 4E). Based on ENCODE database, H3K27ac was predicted to be enriched in the promoters of IL6ST, CTSL, MAD2L1, and FAM3C (Figure 4F; Supplementary Figure S3E). ChIP was further used to explore the enrichment of YEATS2 and H3K27ac in the promoters of genes, and the result identified that both YEATS2 and H3K27ac were significantly enriched in the IL6ST promoter in KYSE150 cells (Figure 4G). And the enrichment of YEATS2 in the promoter of IL6ST was further validated by ChIP-seq (Supplementary Table S7). Therefore, YEATS2 might regulate IL6ST through H3K27ac.

Furthermore, knocking down IL6ST by RNA interference reversed the effect of YEATS2 on ESCC cells (Figure 4H; Supplementary Figure S3F) and its regulation of NF-κB protein level (Figure 4I). IL6ST is a component of cytokine receptor complexes shared by many cytokines, including IL6, CNTF, LIF, and OSM. And it has been reported that NF-κB signaling could be regulated by cytokines such as IL6. As a result, YEATS2 might regulate NF-κB signaling pathway through regulating the expression of IL6ST.

### 3.5 TAF15 and KAT5 participated in YEATS2 regulation of IL6ST

**FIGURE 5:**
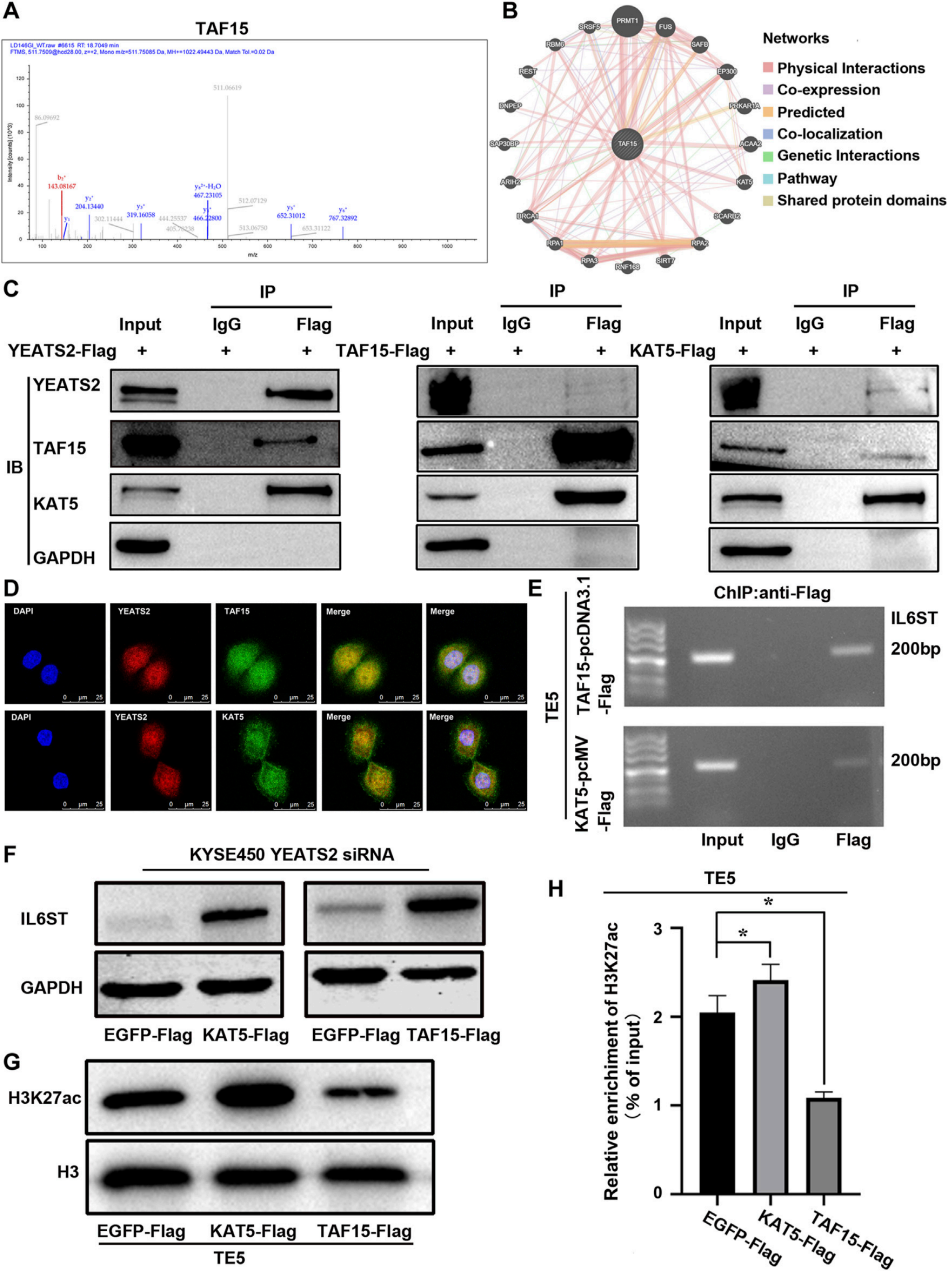
TAF15 and KAT5 participated in the regulation of YEATS2 on H3K27ac and IL6ST. **(A)** Co-IP-based mass spectrum (MS) assays were performed in TE5 cells with YEATS2 overexpression. The second-order spectrum of TAF15 was derived from Co-IP-based MS. **(B)** GeneMANIA database was used to predict the proteins binding to TAF15. **(C)** Co-IP was employed to test interactions among YEATS2, TAF15 and KAT5. **(D)** The subcellular localizations of YEATS2, TAF15 and KAT5 were tested by IF. **(E)** ChIP was employed to test TAF15 and KAT5 enrichment in the IL6ST promoter region. **(F)** KAT5 and TAF15 participated in the regulation of YEATS2 on the protein expression level of IL6ST. **(G)** KAT5 but not TAF15 could increase the level of H3K27ac. **(H)** KAT5 but not TAF15 could increase H3K27ac enrichment in IL6ST promoter, which was verified by ChIP-qPCR. Data shown are the mean ± SD of three biological replicates. *p* values were calculated by unpaired t tests with *p < 0.05, **p < 0.01, and ***p < 0.001.

YEATS2 and H3K27ac were enriched in the promoter of IL6ST, but the molecular mechanism through which YEATS2 recognized and regulated H3K27ac remain to be elucidated. Histone acetylation readers, including BRD domain proteins (Phillips et al., 2024) and YEATS domain proteins (Mi et al., 2017) usually regulate histone acetylation by interacting with histone acetyltransferases. Hence, we further performed Co-IP-based mass spectrum (MS) assays in TE5 cell with YEATS2 overexpression to explore the interacting protein participating in the regulation of YEATS2 and H3K27ac on IL6ST. The intersection of two biological repeats of IP-MS is 37 proteins (Supplementary Table S8). Among the 37 potential YEATS2-binding proteins, TAF15 (Figure 5A) has been predicted to interact with histone acetyltransferase KAT5 using GeneMANIA database (Figure 5B). KAT5 was primarily purified as a catalytic subunit of the NuA4 histone acetyltransferase complex and harbored HAT activity towards histone H4 and H2A, but an increasing number of studies suggests that KAT5 could be involved in transcriptional activation of select genes by acetylation of histone H3K27 (Frank et al., 2003; Taubert et al., 2004; Luo et al., 2022). Consequently, TAF15, a component of transcription initiation factor TFIID complexes, might participate in the regulation of YEATS2 and H3K27ac on IL6ST through interacting with YEATS2, which was proved by Co-IP assay (Figure 5C). And histone acetyltransferase KAT5 can not only interact with TAF15 but also with YEATS2 in TE5 cells, which were also verified by Co-IP assay (Figure 5C). The results of IF assay further showed that YEATS2, TAF15 and KAT5 located in the KYSE150 cell nucleus, which suggested a possible colocalization among them (Figure 5D). Therefore, YEATS2 might bind to TAF15 and KAT5 simultaneously in the ESCC cell nucleus.

Whether TAF15 and KAT5 are involved in the regulation of YEATS2 on H3K27ac and IL6ST need further investigation. Considering that YEATS2 and H3K27ac were enriched in the IL6ST promoter, and YEATS2 could bind to TAF15 and KAT5. ChIP was further used to detect the enrichment of TAF15 and KAT5 in IL6ST promoter. Like YEATS2 and H3K27ac, TAF15 and KAT5 were also enriched in the IL6ST promoter in TE5 cell (Figure 5E). And both KAT5 and TAF15 could weaken the suppressive effect of YEATS2 knockdown on IL6ST protein expression in KYSE450 cell (Figure 5F). Consequently, TAF15 and KAT5 might be involved in the regulation of YEATS2 on IL6ST. The results of Western blot further showed that KAT5, but not TAF15, could increase the level of H3K27ac in TE5 cells (Figure 5G). Meanwhile KAT5 could increase the H3K27ac enrichment in IL6ST promoters in TE5 cells, which was clarified by ChIP-qPCR (Figure 5H).

**FIGURE 6:**
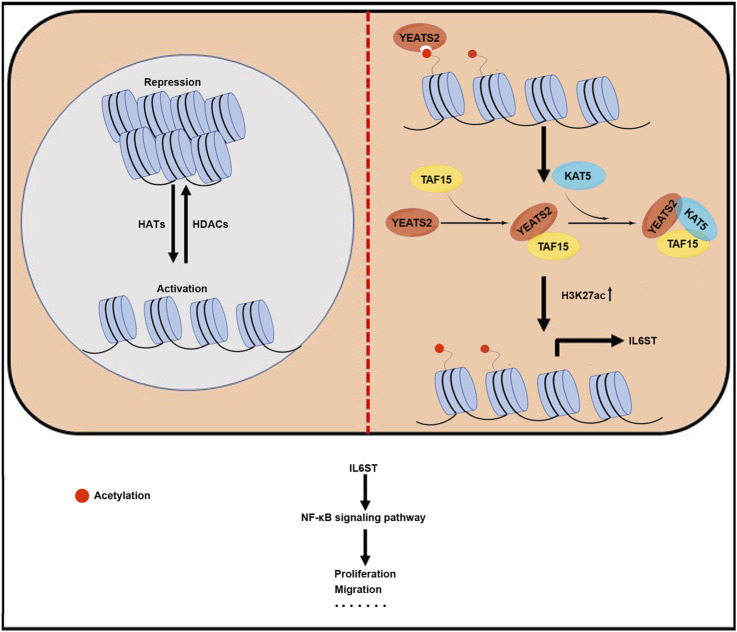
A proposed working model for YEATS2/TAF15/KAT5 complex regulating NF-κB by H3K27 acetylation activated-IL6ST in ESCC.

Consequently, YEATS2 might recruit KAT5 to IL6ST promoter through TAF15, and KAT5 increased the H3K27ac enrichment in IL6ST promoters and promoted the transcription of IL6ST (Figure 6).

## 4 Discussion

We have explored the clinical significance, function, and molecular mechanism of YEATS2. YEATS2, a significantly mutated and amplified gene, was related to the patient differentiation and prognosis in ESCC. And *in vivo* and *in vitro* experiments showed that YEATS2 increased the abilities of ESCC cells to proliferate and migrate. Mechanistically, YEATS2 recruited KAT5 through TAF15, which in turn enhanced H3K27ac enrichment in the IL6ST promoter region and ultimately activated NF-κB signaling pathway in ESCC.

According to previous studies, YEATS2 could play a carcinogenic role in pancreatic ductal adenocarcinoma (Sheng et al., 2023), pancreatic cancer (Zeng et al., 2021), and non-small cell lung cancer (Mi et al., 2017). However, YEATS2 was first reported as a significantly mutated gene and its function was not reported in ESCC (Cui et al., 2020). WGS analysis showed that YEATS2 not only had high-frequency mutations but also copy number amplification in ESCC tissues. Besides, YEATS2 was significantly upregulated in ESCC tissues. And YEATS2 was associated with the differentiation degree of ESCC cells and the postoperative survival of ESCC patients. These results support the idea that YEATS2 might play a carcinogenic role in ESCC. However, detection of YEATS2 expression levels based on the IHC is further needed to validate the clinical significance of YEATS2.

*In vivo* and *in vitro* experiments supported the tumor promoting effect of YEATS2. According to RNA-seq and KEGG analysis, the DEGs induced by YEATS2 knockdown were mainly enriched in NF-κB signaling pathway, which could regulate multiple tumors development processes through various mechanisms. And we have found YEATS2 could regulate NF-κB signaling pathway and activating or inhibiting NF-κB signaling pathway could rescue YEATS2 effect on ESCC cells.

YEATS domain proteins, a kind of histone acetylation readers, interact with HAT complexes and transcription-regulating complexes (Schulze et al., 2009). And YEATS domain proteins could regulate the occurrence and development of multiple tumors by participating in histone acetylation and gene transcription. For example, YEATS1 co-located with H3K9ac and H3K27ac on the promoter region of active transcription genes necessary for leukemia and promoted the progression of AML (Wan et al., 2017). YEATS2 regulated H3K27ac and H3K9ac in NSCLC and promoted the progression of NSCLC (Mi et al., 2017). YEATS4 co-located with H3K14 acetylation (H3K14ac) and H3K27ac on the promoter region of active transcription genes and promoted the progression of NSCLC (Hsu et al., 2018). Whether YEATS2 regulation on NF-κB signaling pathway in ESCC involves histone acetylation, chromatin remodeling and gene transcription need to be further explored.

We further found that YEATS2 could regulate exclusively H3K27ac in ESCC cells. YEATS2, a scaffolding subunit of the ATAC complex, binds specifically to histone H3K27ac in NSCLC. And it recruits the ATAC complex to H3K27ac-enriched target genes to promote active transcription via maintaining promoter histone H3K9ac level. YEATS2, meanwhile, regulates the levels of H3K27ac and H3K9ac through the HAT module of ATAC complex (Mi et al., 2017). But in our present study, we have found that YEATS2 regulated H3K27ac exclusively and had no effect on the level of H3K9ac in ESCC. Therefore, we speculated that YEATS2, a histone H3K27ac reader, might exert a regulatory effect on H3K27ac by recruiting other acetyltransferases in ESCC. H3K27ac enrichment in the promoter region of genes is a transcription activation marker. And both YEATS2 and H3K27ac were enriched in IL6ST promoter. IL6ST is a component of cytokine IL6 receptor complexes. And it has been reported that NF-κB signaling could be regulated by cytokine IL6 (Kim et al., 2022; Liu et al., 2018). We have also found that IL6ST could mediate the effect of YEATS2 on ESCC cells and NF-κB signaling pathway. As a signal transducer, IL6ST could regulate multiple tumors development processes including head and neck squamous cell carcinoma (Sriuranpong et al., 2003), bladder cancer (He et al., 2021), hepatocellular carcinoma (Desideri et al., 2023), gastric cancer (Chen et al., 2020), colorectal cancer (Zhao et al., 2024), breast cancer (Wang et al., 2020), prostate cancer (Sternberg et al., 2024). Therefore, we suspect that upregulation of IL6ST induced by YEATS2-mediated H3K27ac activated NF-κB signaling pathway.

YEATS domain proteins can be involved in the regulation of histone acetylation through acetyltransferase. Thereby, we performed Co-IP-based MS assays to detect the proteins through which YEATS2 can regulate H3K27ac and IL6ST in ESCC cells. TAF15 was found to interact with YEATS2, and this interaction was verified by Co-IP and IF. TAF15, a critical component of TFIID complex, functions in RNA polymerase II gene transcription (Yang et al., 2023). Therefore, TAF15 might participate in the transcriptional regulation of IL6ST by YEATS2. The balance of histone acetylation level in physiological state is maintained by histone acetyltransferase and deacetylase. KAT5, a histone acetyltransferase and a catalytic subunit of the NuA4 histone acetyltransferase complex, could be involved in transcriptional activation of select genes by acetylation of histone H3 lysine 27. KAT5 was further found to interact with TAF15 and YEATS2. KAT5 and TAF15 were both enriched in IL6ST promoter and participated in the regulation of IL6ST by YEATS2, but only KAT5 increased the enrichment degree of H3K27ac in IL6ST promoter. However, as a key subunit of transcription initiation complex, TAF15 may regulate the expression of IL6ST through other molecular mechanisms that need further exploration. Consequently, YEATS2 could further recruit TAF15 and KAT5 to enhance the enrichment of H3K27ac in the IL6ST promoter. Furthermore, they increased the expression of IL6ST and activated NF-κB signaling pathway to promote ESCC progression.

## 5 Conclusion

In conclusion, we have proved that the expression and copy number variation of YEATS2 were related to the tumor differentiation and the prognosis of patients in ESCC. And YEATS2/TAF15/KAT5 complex regulated NF-κB signaling pathway via H3K27ac activated-IL6ST in ESCC. Consequently, we speculate that inhibitors of histone acetylation readers targeting YEATS2 would be effective treatment strategies for ESCC.

## Data Availability

The datasets presented in this study can be found in online repositories. The names of the repository/repositories and accession number(s) can be found in the article/Supplementary Material.

## Abbreviations

CNV, Copy numer variation; CNA, Copy number amplification; Co-IP, Co-Immunoprecipitation; ChIP, Chromatin Immunoprecipitation; EAC, Esophageal Adenocarcinoma; ESCC, Esophageal Squamous Cell Carcinoma; H3, H3 histone; H3K9ac, H3K9 acetylation; H3K14ac, H3K14 acetylation; H3K18ac, H3K18 acetylation; H3K27ac, H3K27 acetylation; IF, Immunofluorescence; IHC, Immunohistochemistry; IL6ST, Interleukin 6 cytokine family signal transducer; KAT5, Lysine acetyltransferase 5; NF-κB, Nuclear factor kappa B; RNA-seq, RNA-sequencing; RT-qPCR, Real-time quantitative PCR; siRNA, Small interference RNA; TAF15, TATA-box binding protein associated factor 15; WES, Whole exome sequencing; WGS, Whole genome sequencing; YEATS2, YEATS domain containing 2.
