# Supplementary figures and images for: YEATS2 promotes malignant phenotypes of esophageal squamous cell carcinoma via H3K27ac activated-IL6ST
Source: Front Cell Dev Biol. 2025 Feb 18;13:1497290. doi: 10.3389/fcell.2025.1497290 (PMC11876388; doi:10.3389/fcell.2025.1497290)

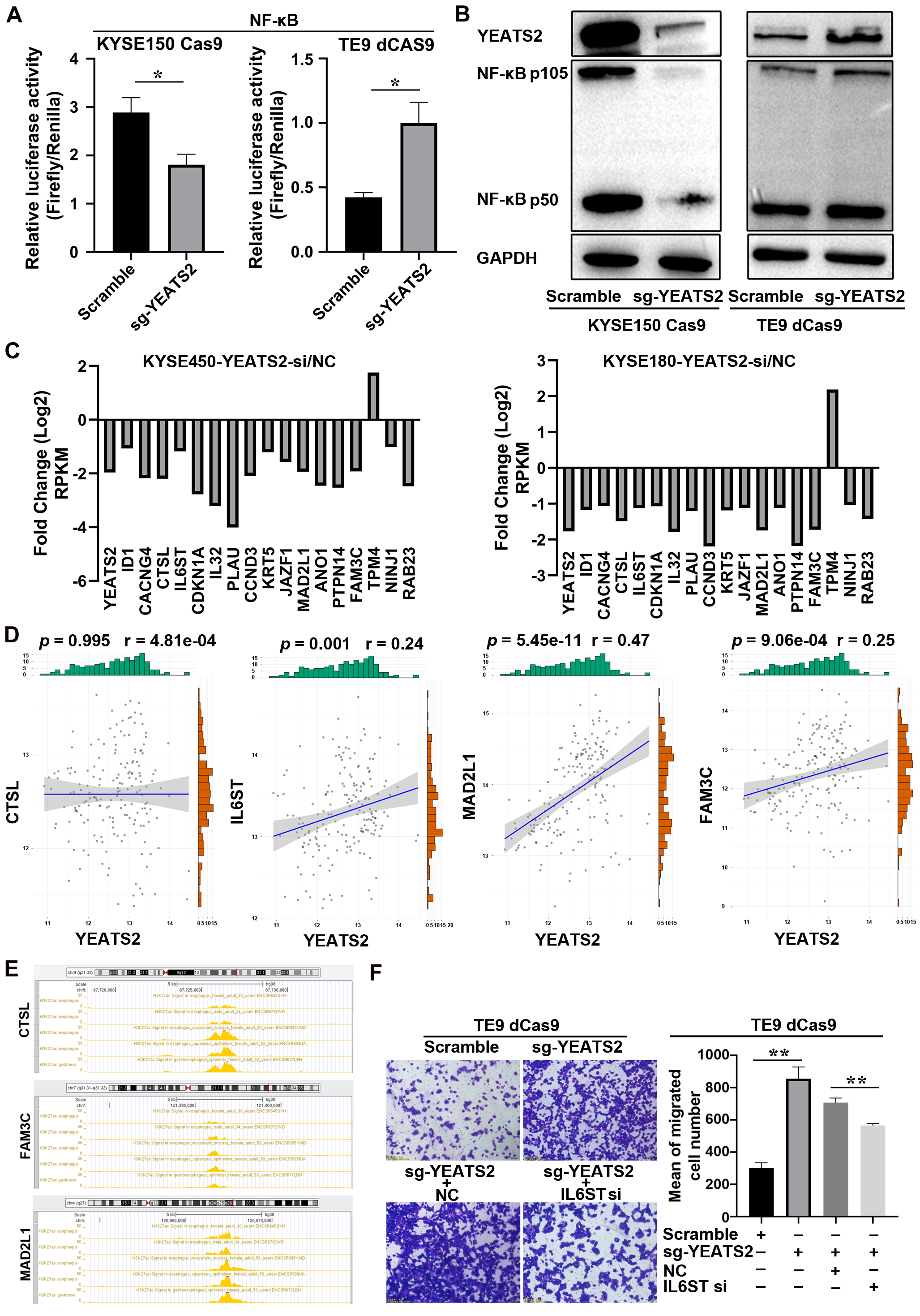

Supplement: Supplementary file 3 [file Image3.tif]

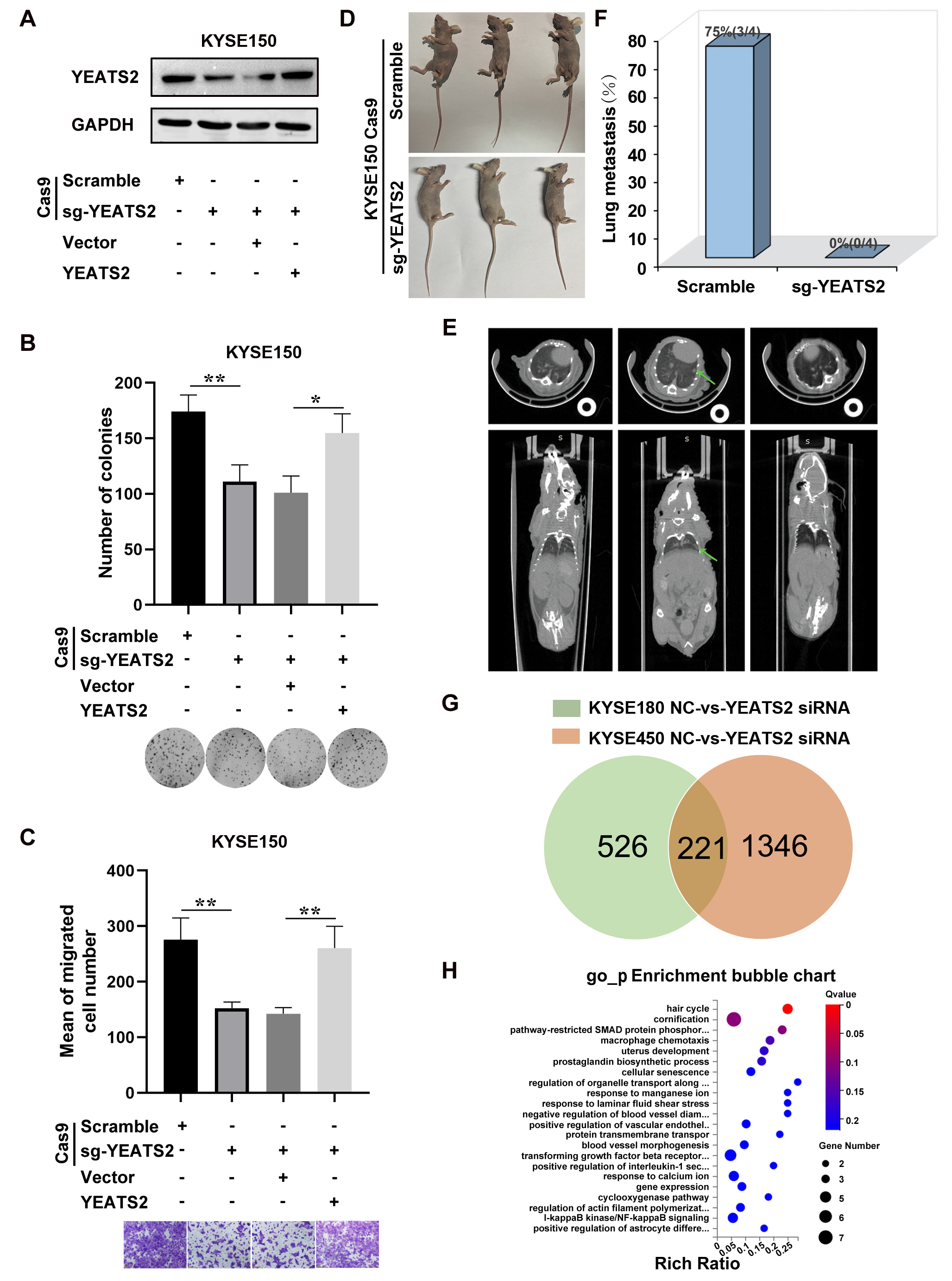

Supplement: Supplementary file 4 [file Image2.tif]

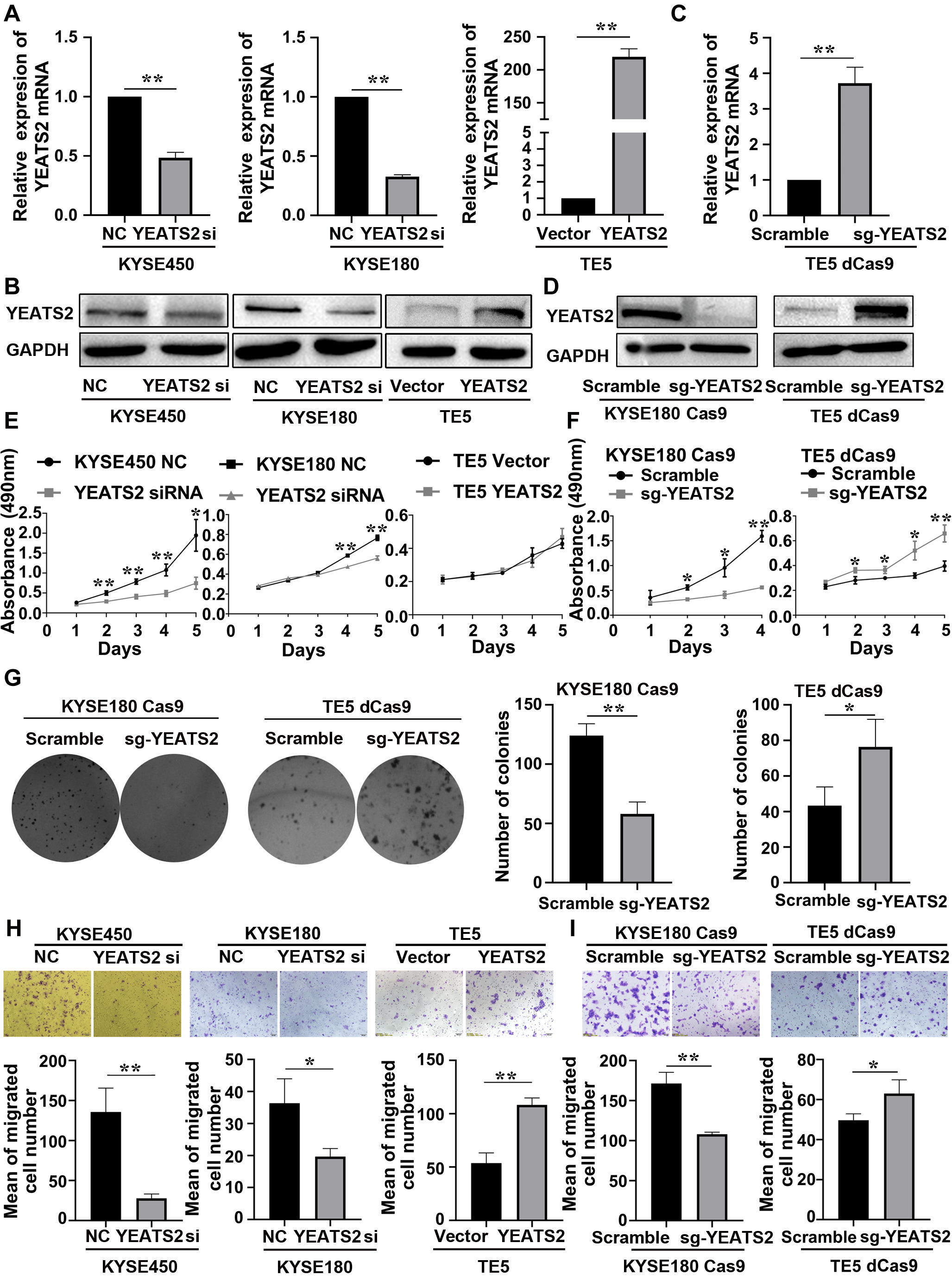

Supplement: Supplementary file 5 [file Image1.tif]
