# Supplementary material for: YEATS2 promotes malignant phenotypes of esophageal squamous cell carcinoma via H3K27ac activated-IL6ST
Source: Front Cell Dev Biol. 2025 Feb 18;13:1497290. doi: 10.3389/fcell.2025.1497290 (PMC11876388; doi:10.3389/fcell.2025.1497290)
Supplement: Supplementary file 12 [file DataSheet1.docx]

**Supplementary figure legends**

**Fig. S1** **YEATS2 knockdown inhibited** **the abilities of ESCC cells to proliferate and migrate *in vitro*.**

**(A-B)** We performed RT-qPCR and Western Blot to verify the efficiency of YEATS2 knockdown and overexpression acted by siRNAs and overexpressed plasmids. Data shown are the mean ± SD of three technical replicates. *p* values were calculated by unpaired t tests with **p* < 0.05, ***p* < 0.01, and ****p* < 0.001. **(C-D)** We performed RT-qPCR and Western Blot to verify the efficiency of YEATS2 knockdown and overexpression acted by CRISPR/Cas9 and CRISPR/dCas9-SAM system. Data shown are the mean ± SD of three technical replicates. *p* values were calculated by unpaired t tests with **p* < 0.05, ***p* < 0.01, and ****p* < 0.001. **(E-F)** MTT was used to detect YEATS2 effect on the ESCC cells abilities to proliferate *in vitro*. Data shown are the mean ± SD of three technical replicates. *p* values were calculated by unpaired t tests with **p* < 0.05, ***p* < 0.01, and ****p* < 0.001. **(G)** Colony Formation Assay was performed to detect YEATS2 effect on the colony forming ability of ESCC cells *in vitro*. Data shown are the mean ± SD of three technical replicates. *p* values were calculated by unpaired t tests with **p* < 0.05, ***p* < 0.01, and ****p* < 0.001. **(H-I)** Transwell was used to detect YEATS2 effect on the ESCC cells abilities to migrate *in vitro*. Bar graphs (bottom panel) display the number of migrated cells. Data shown are the mean ± SD of three technical replicates. *p* values were calculated by unpaired t tests with **p* < 0.05, ***p* < 0.01, and ****p* < 0.001.

**Fig. S2 (A)** Western Blot was performed to verify the efficiency of YEATS2 knockdown and overexpression in KYSE150. **(B)** Colony Formation Assay was performed to detect YEATS2 effect on the colony forming ability of ESCC cells in KYSE150. Data shown are the mean ± SD of three biological replicates. *p* values were calculated by unpaired t tests with **p* < 0.05, ***p* < 0.01, and ****p* < 0.001. **(C)** Transwell was used to detect YEATS2 effect on the migration ability of ESCC cells in KYSE150 and TE9. Data shown are the mean ± SD of three biological replicates. *p* values were calculated by unpaired t tests with **p* < 0.05, ***p* < 0.01, and ****p* < 0.001.

**(D)** Photographic images of tumor-bearing nude mice (n = 3 in each group). **(E)** CT images for nude mice of tail-vein lung metastatic models. **(F)** Statistics of lung metastasis in each group. Bar graphs display the lung metastasis rate in the in scramble or sg-YEATS2 groups (n = 4 in each group). **(G)** The numbers of DEGs in KYSE180 and KYSE450 after YEATS2 knockdown from RNA-seq. **(H)** GO terms in the GO enrichment analysis of DEGs in biological process categories with the 22 lowest P values.

**Fig. S3 (A)** The YEATS2 regulations on the transcriptional activity of NF-κB signaling pathway were verified by Dual-Luciferase Reporter Assay. Data shown are the mean ± SD of three technical replicates per cell line. *p* values were calculated by unpaired t tests with **p* < 0.05, ***p* < 0.01, and ****p* < 0.001. **(B)** The YEATS2 regulations on NF-κB p105 and p50 protein levels were verified by Western Blot. **(C)** The data of mRNA expression levels of YEATS2 and 17 DEGs were collected from RNA-seq. **(D)** The mRNA expression level correlations of YEATS2 with IL6ST, MAD2L1, FAM3C and CTSL were analysed by using TCGA GSE53625 data. Statistical significance was assessed by Pearson's chi-squared test. **(E)** The ENCODE was used to predict H3K27ac enrichment in the promoter of CTSL, FAM3C and MAD2L1. **(F)** IL6ST knockdown partially reversed the effect of YEATS2 on the migration of TE9. Data shown are the mean ± SD of three technical replicates. *p* values were calculated by unpaired t tests with **p* < 0.05, ***p* < 0.01, and ****p* < 0.001.
